# Synergism in Antiplasmodial Activities of Artemether and Lumefantrine in Combination with *Securidaca longipedunculata* Fresen (Polygalaceae)

**DOI:** 10.3390/plants11010047

**Published:** 2021-12-24

**Authors:** Douglas O. Ochora, Esezah K. Kakudidi, Jane Namukobe, Perpetua Ipulet, Dancan M. Wakoli, Winnie Okore, Edwin W. Mwakio, Redempthah A. Yeda, Agnes C. Cheruiyot, Dennis W. Juma, Ben Andagalu, Amanda L. Roth, Bernhards R. Ogutu, Abiy Yenesew, Hoseah M. Akala

**Affiliations:** 1 Department of Plant Sciences, Microbiology & Biotechnology, College of Natural Sciences, Makerere University, Kampala P.O. Box 7062-10207, Uganda; ekakudidi@cns.mak.ac.ug (E.K.K.); perpetua.ipulet@mak.ac.ug (P.I.); 2 Department of Chemistry, College of Natural Sciences, Makerere University, Kampala P.O. Box 7062-10207, Uganda; jane.namukobe@mak.ac.ug; 3 Department of Biochemistry and Molecular Biology, Egerton University, Njoro P.O. Box 536-20115, Kenya; Duncan.Wakoli@usamru-k.org; 4 United States Army Medical Research Directorate-Kenya (USAMRD-K), Kenya Medical Research Institute (KEMRI)—Walter Reed Project, Kisumu, Kisumu P.O. Box 54-40100, Kenya; winnie.okore@usamru-k.org; 5 Department of Biomedical Sciences and Technology, School of Public Health and Community Development, Maseno University, Maseno P.O. Box Private Bag-40105, Kenya; Edwin.Mwakio@usamru-k.org (E.W.M.); Redemptah.Yeda@usamru-k.org (R.A.Y.); Agnes.Cheruiyot@usamru-k.org (A.C.C.); Dennis.Juma@usamru-k.org (D.W.J.); Ben.Andagalu@usamru-k.org (B.A.); amanda.roth@usamru-k.org (A.L.R.); 6 Centre for Clinical Research, Kenya Medical Research Institute (KEMRI), Kisumu P.O. Box 1578-40100, Kenya; ogutu6@gmail.com; 7 Department of Chemistry, University of Nairobi, Nairobi P.O. Box 30197-00100, Kenya; ayenesew@uonbi.ac.ke

**Keywords:** combination, malaria, plasmodium falciparum, *Securidaca longipedunculata*, synergism

## Abstract

Malaria is the most lethal parasitic disease in the world. The frequent emergence of resistance by malaria parasites to any drug is the hallmark of sustained malaria burden. Since the deployment of artemisinin-based combination therapies (ACTs) it is clear that for a sustained fight against malaria, drug combination is one of the strategies toward malaria elimination. In Sub-Saharan Africa where malaria prevalence is the highest, the identification of plants with a novel mechanism of action that is devoid of cross-resistance is a feasible strategy in drug combination therapy. Thus, artemether and lumefantrine were separately combined and tested with extracts of *Securidaca longipedunculata*, a plant widely used to treat malaria, at fixed extract–drug ratios of 4:1, 3:1, 1:1, 1:2, 1:3, and 1:4. These combinations were tested for antiplasmodial activity against three strains of *Plasmodium falciparum* (W2, D6, and DD2), and seven field isolates that were characterized for molecular and ex vivo drug resistance profiles. The mean sum of fifty-percent fractional inhibition concentration (FIC_50_) of each combination and singly was determined. Synergism was observed across all fixed doses when roots extracts were combined with artemether against D6 strain (FIC_50_ 0.403 ± 0.068) and stems extract combined with lumefantrine against DD2 strain (FIC_50_ 0.376 ± 0.096) as well as field isolates (FIC_50_ 0.656 ± 0.067). Similarly, synergism was observed in all ratios when leaves extract were combined with lumefantrine against W2 strain (FIC_50_ 0.456 ± 0.165). Synergism was observed in most combinations indicating the potential use of *S. longipedunculata* in combination with artemether and lumefantrine in combating resistance.

## 1. Introduction

Fear of malaria among individuals residing, and visiting endemic regions has been sustained by the continuous malaria infection causing over 1000 deaths daily in 2019, globally [1]. The situation has worsened by the emergence and spread of resistant *Plasmodium falciparum* strains to all standard antimalarial drugs [2]. The spread of resistant *P. falciparum* strains is facilitated by high gametocyte production in partially resistant strains and exacerbated by population movement and globalization [3]. Resistance of *P. falciparum* to antimalarial drugs such as chloroquine, quinine, mefloquine, halofantrine, and artemisinin-based combination therapies (ACTs), the recommended treatment for uncomplicated malaria has been reported [4,5,6], warrants the search for new drugs and combinations with unique mechanisms of action.

In the 20th century, the development of resistant strains of infectious diseases such as leprosy [7], tuberculosis [8], and human immunodeficiency virus [9] has led to the development of combination therapy as the standard treatment for these and other diseases, which is also recommended for treatment of malaria [10]. Consequently, the global malaria burden caused by *P. falciparum* was reduced by the use of artemisinin-based combination therapies (ACTs) including artemether-lumefantrine, dihydroartemisinin-piperaquine, artesunate-amodiaquine, and artesunate-mefloquine [11]. Artemether-lumefantrine was the first co-formulated ACTs and the most widely used first-line ACT in the treatment of uncomplicated falciparum malaria in sub-Saharan Africa [12]. However, the increasing reports on resistance to ACT necessitate reciprocal innovation of new drugs with different mechanisms of action. In sub-Saharan Africa, where there is a high prevalence of malaria, a combination of traditionally used antimalarial plants with known antimalarial drugs could be a practical strategy to combat the disease.

Traditional antimalarial medicinal plants remain sources of drugs with unique structures. The medicinal plants potentially have a different mode of action as depicted by the success of artemisinin, isolated from the *Artemisia annua* L. (Asteraceae) and quinine from *Cinchona* species (Rubiacieae) [5,13]. In the quest to identify and utilize plants with similar use, this study focused on *Securidaca longipedunculata* Fresen, (Family Polygalaceae) in combination with some standard drugs. *S. longipedunculata* has been used as a remedy for various disease conditions in traditional medicine [14], including malaria in Kenya [15], Uganda [16,17], Tanzania [18], Burkina Faso [19], and Nigeria [20]. In a previous study, we have reported that the methanol extract of the roots of *S. longipedunculata* showed antiplasmodial activity with an IC_50_ value of 1.4 ± 0.07 μg/mL against the W2 strain of *P. falciparum* [21]. Notably, people in malaria-endemic areas are likely to take herbal medicine before seeking treatment in the formal health sector for the recommended antimalarial ACTs [22]. Using reference strains and field isolates obtained from individuals with naturally acquired infections for field expedience, we herein report the in vitro antiplasmodial test results of the combination of the extracts of the roots, stems, and leaves of *S. longipedunculata* with artemether and lumefantrine against *P. falciparum* strains.

## 2. Results

### 2.1. Antiplasmodial Activities

The in vitro and ex vivo test results of the methanol extracts from different plant parts of *S. longipedunculata* are given in Table 1. The roots extract was the most active of the three extracts against the field isolates ex vivo and the reference strains followed by leaves extract while stems extract was the least active (Table 1). These extracts and reference drugs namely artemether, lumefantrine, mefloquine, and chloroquine were more active against the reference strains D6, W2, and DD2 of *P. falciparum* in vitro than the field isolates ex vivo. The D6 strain was more susceptible to chloroquine while W2 strain had IC_50_ greater than 45 ng/mL depicting resistance.

The results of the assessment of response of the field isolates and reference strains to extracts of *S. longipedunculata* in combination with either artemether or lumefantrine revealed diverse responses ranging from synergism, additivity to antagonism. All combination tests that were done against the reference strains namely W2: chloroquine-resistant, mefloquine sensitive; DD2: chloroquine-resistant, mefloquine resistant and D6: chloroquine-sensitive, mefloquine resistant strains of *P. falciparum* resulted in significant parasite growth inhibition (*p* < 0.05). The mean sum of fifty-percent fractional inhibition concentration (FIC_50_) is grouped as either synergism (FIC_50_ < 1), additivity (FIC_50_ = 1), or antagonism (FIC_50_ > 1).

**Figure 1:**
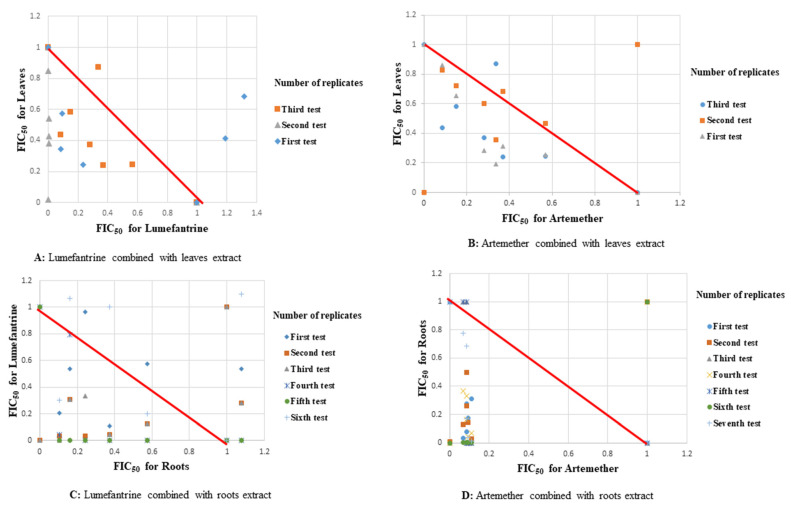
Scatter plots of drugs-extracts combinations against W2 strain of *P. falciparum*.

**Figure 2:**
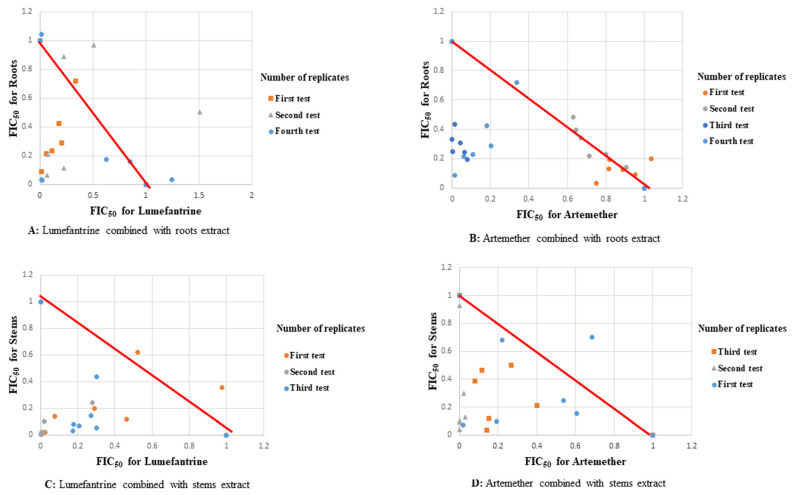
Scatter plots of drugs-extracts combinations against DD2 strain of *P. falciparum*.

Synergism was observed in all fixed ratio combinations of lumefantrine-leaves extract against W2 strain of *P. falciparum.* At a fixed ratio of 1:2, the sum FIC_50_ value was 0.262 ± 0.122 (Table 2, Figure 1). All combinations of the artemether-stems extract, lumefantrine-stems extract and lumefantrine-roots extract showed synergism when tested against the DD2 strain (Table 2, Figure 2). Synergism of the artemether-stems extract combination against this strain at a fixed ratio of 3:1 (drug:extract) had a mean sum FIC_50_ value of 0.219 ± 0.082 (Table 2, Figure 2).

**Figure 3:**
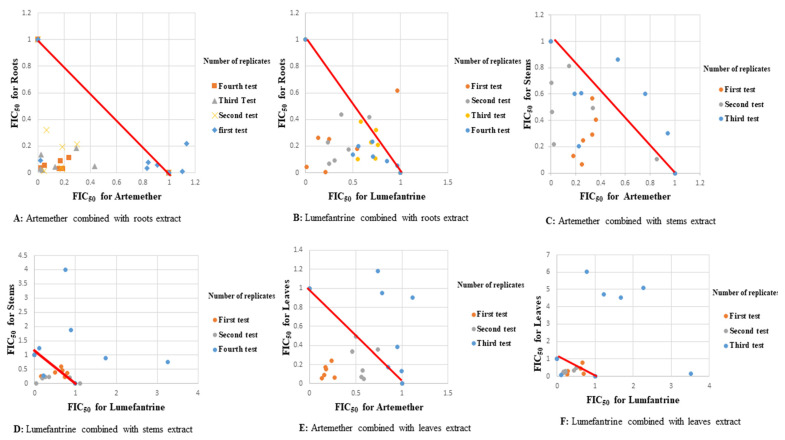
Scatter plots of drug-extract combinations against D6 strain of *P. falciparum*.

All fixed ratios of artemether-roots extract, artemether-stems extract, and lumefantrine-roots extract combinations showed synergism when tested against D6 strain of *P. falciparum*. This synergism was mainly observed at a fixed ratio of 1:4 (drug:extract) with a mean sum FIC_50_ value of 0.284 ± 0.159 (Table 3). All roots extract combinations showed synergism against the D6 strain (Table 3, Figure 3).

**Figure 4:**
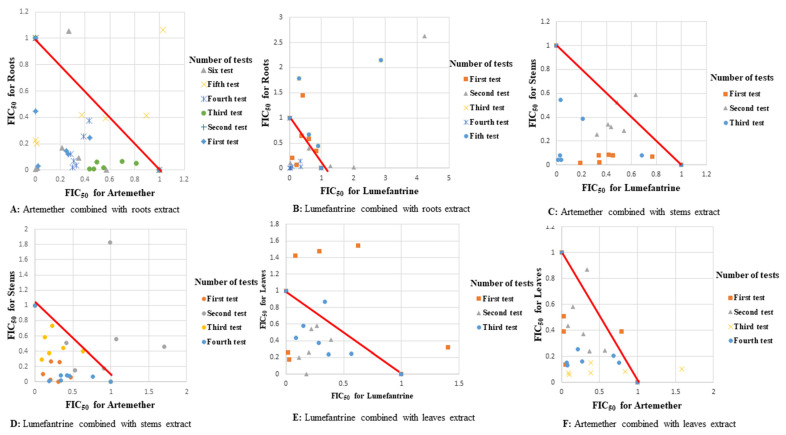
Scatter plots of drugs-extracts combinations against field isolates ex vivo of *P. falciparum*.

Synergism was observed at all fixed ratios of artemether-stems extract when tested against field isolates ex vivo (Table 4). The artemether-leaves extract combination at fixed ratio of 3:1 also showed synergism with a mean sum FIC_50_ value of 0.289 ± 0.155 against field isolates ex vivo (Table 4). Similar synergistic responses were observed at all the fixed doses of artemether-roots extract and lumefantrine-stems extract combinations against field isolates ex vivo except at a fixed ratios of 1:4 and 1:1 respectively which showed antagonism (Figure 4).

**Figure 5:**
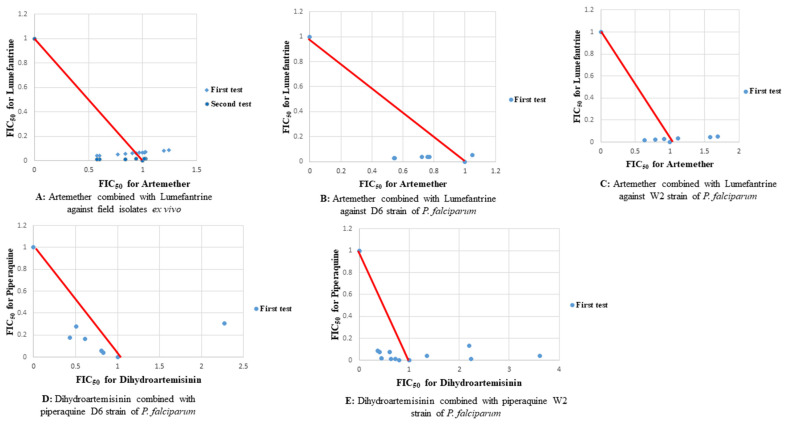
Scatter plots of control drugs combinations.

Assessment of two of the recommended artemisinin-based combinations at same fixed dose ratio matrices revealed varying responses. Synergism was observed at most fixed dose ratios of artemether-lumefantrine and dihydroartemisinin-piperaquine combinations against field isolates, reference strains of *P. falciparum* D6 and W2 as well as D6 and W2 (Figure 5).

Responses of extracts of *S. longipedunculata* in combination with either artemether or lumefantrine against reference strains and field isolates are summarized in Table 5. Synergism was observed at all fixed ratios of lumefantrine-roots extract and artemether-roots extract combinations with a mean sum FIC_50_ values of 0.75 ± 0.207 and 0.856 ± 0.448 respectively, both tested against W2 strain (Table 5). Synergism was also observed across all fixed ratios of lumefantrine-leaves extract combination with a mean sum FIC_50_ value of 0.456 ± 0.165 against W2 strain of *P. falciparum* (Table 5, Figure 1). Similarly, synergism was observed across all fixed ratios of the lumefantrine-stems extract combination with a mean sum FIC_50_ of 0.376 ± 0.096; artemether-stems extract combination with a mean sum FIC_50_ of 0.485 ± 0.176, lumefantrine-roots extract combination with a mean sum FIC_50_ of 0.629 ± 0.18; and all combinations tested against DD2 strain (Table 5, Figure 2).

Synergism was observed across all fixed doses of artemether-roots extract of *S. longipedunculata*, against the D6 strain of *P. falciparum* with a mean sum FIC_50_ of 0.403 ± 0.068 (Table 5). Synergism was also observed across all fixed doses of lumefantrine-roots extract and artemether-stems extract combinations with mean sum FIC_50_ of 0.738 ± 0.093 and mean sum FIC_50_ of 0.756 ± 0.126 against D6 strain of *P. falciparum,* respectively (Table 5). Synergism of all tests against field isolates ex vivo was especially observed across all fixed ratios of artemether-stems extract combination with a mean sum FIC_50_ of 0.656 ± 0.067 (Table 5). A similar synergistic response was observed at all fixed ratios of artemether-leaves extract combination at a mean sum FIC_50_ of 0.705 ± 0.398 and lumefantrine-leaves extract combination with a mean sum FIC_50_ of 0.825 ± 0.252 both tested against field isolates ex vivo (Table 5). All the fixed doses of artemether-roots extract and lumefantrine-stems extract combinations were synergistic, giving mean sum FIC_50_s of 0.805 ± 0.229 and 0.807 ± 0.218 respectively against field isolates ex vivo (Table 5, Figure 4).

### 2.2. Molecular Analysis

Analyses of the deoxyribonucleic acid (DNA) extracted from each of the field isolate tested for ex vivo antiplasmodial activity revealed that all field isolates tested positive for *P. falciparum.* (Table S3). Single nucleotide polymorphisms (SNPs) analyses of putative drug resistance genes showed that all the isolates carried a mutant *P. falciparum* dihydrofolate reductase *Pfdhfr* 59C genotype. Other mutant genotypes were detected in *P. falciparum* multidrug resistance protein 1 (*Pfmrp* 1 876G) in three field isolates and *P. falciparum* multidrug resistance gene 1 (*Pfmdr* 1 184T) in two isolates. The wild-type genotypes were observed in *P. falciparum* dihydropteroate synthetase (*Pfdhps* 437G) in all field isolates. A mixed infection consisting of both wild-type and mutant was observed in *Pfmdr* 1 184A/T (one isolate) and *Pfmrp* 1 G/A (three isolates) (Table S3).

## 3. Discussion

In vitro antiplasmodial activities of drug combinations were done to determine which combinations would be clinically effective for the treatment of malaria. Combining two drugs that have different mechanisms of action is likely to reduce the emergence of drug-resistant strains. Furthermore, in combination treatment, synergism often results in the use of a lower amount of each drug [23,24], lessening the cumulative drug pressure in the population, therefore, reducing the pace of emergence of resistance to either of the drugs. Ex vivo and in vitro antiplasmodial assays revealed synergism between artemether or lumefantrine (the principal components of the Coartem^®^ which is the recommended first-line drug for the treatment of uncomplicated malaria) in combination with *S. longipedunculata* extracts, a plant species traditionally used as an antimalarial remedy in many countries across Africa. 

Synergism between standard antimalarial drugs and plant extracts from medicinal plants used traditionally for the treatment of malaria by the Meru community in Kenya has previously been reported [25]. Other studies on combination between standard antimalarial drugs and crude extracts have reported similar findings, where aqueous leaves extract of *Telfaria occidentalis* (200 mg/kg) combined with artesunate (2 mg/kg) showed modest synergism of 85.43% against *Plasmodium berghei* malaria model [26]. In another similar study, aqueous leaves extract of *Ageratum conyzoides* potentiated the antiplasmodial activities of chloroquine and artesunate against *P. berghei* in the albino mice model [27]. This report of synergism between standard antimalaria drugs in combination with plant extracts against strains and well-characterized field isolates from naturally acquired infections suggest beneficial outcomes of widespread parallel use of traditional medicine and standard treatment [28] that could play a role in reducing the rate of emergence of resistance. 

The ex vivo and in vitro antiplasmodial activity tests in this study showed synergism in most combination assays. Extracts of *S. longipedunculata* potentiated antiplasmodial activities of lumefantrine against field isolates obtained from individuals with naturally acquired infections as well as *P. falciparum* reference strains (D6, W2, and DD2). These extracts-drug combinations had similar anti-plasmodium activity with widely used ACTs (Figure 5) therefore showing the potential use of the plant in malaria treatment in combination therapy. Notably, there was no difference in response of the field isolates to the varying combinations despite the differences in single nucleotide polymorphisms. These results concur with the wide traditional usage of *S. longipedunculata* in malaria treatment especially the roots [14]. The presence of a variety of phytochemicals in the roots [14] appears to be correlated with the high antiplasmodial activities of the roots. Some of these phytochemicals isolated from the roots of the plant such as 2,3,4,5-tetramethoxybenzophenone, 4-hydroxy-2,3-dimethoxybenzophenone, 3-hydroxy-5-methoxybiphenyl, methyl-2-hydroxy-6-methoxybenzoate, benzyl-2-hydroxy-6-methoxybenzoate, 2-hydroxy-6-methoxybenzoic acid, 2,4,5-trimethoxybenzophenone, and 2-methoxy-(3,4-methylenedioxy)benzophenone were previously tested against *P. falciparum* and showed IC_50_ values ranging from 18.6 μM to 100 μM [21]. Most of the combinations showed synergism though a few showed additivity and antagonism (Table 5). Similar findings were observed when we tested two artemisinin-based combination drugs that are recommended as first line drugs for treatment of uncomplicated malaria in Kenya, at similar fixed ratios. This therefore suggests the potential of the *S. longipedunculata* in treatment of malaria if used alongside these drug combinations. 

In vitro studies using mice models have shown that leaves and stems extracts of the plant are safe at 2000 mg/kg and roots extract is safe at 300 mg/kg [15]. Cytotoxicity assays using normal human cell lines have also showed that pure compounds and extracts from the plant are not toxic to LO2 (liver) and BEAS (lung) normal cells (IC_50_ > 100 μM) [21].

In light of the high in vivo antimalarial activity of extracts and in vitro activity of the pure compounds from the species as previously reported [15,21], and the present results of good synergetic activities against reference strains and characterized field isolates, it can be suggested that the artemether and lumefantrine each combined with *S. longipedunculata* extracts have a good potential in malaria treatment in Africa; the continent carries the brunt of the disease. Overall, the results of the present study serve as additional evidence of the potential use of herb-drug combinations in the treatment of malaria [29], counteracting the ever-increasing emergence of resistant strains of malaria-causing parasites.

## 4. Materials and Methods

### 4.1. Collection of Plant Materials

Roots, leaves, and stems of *Securidaca longipedunculata* were collected from Kwale County, Ukunda Sub-County in Kenya as earlier described [21], where the plant is used in traditional medicine for the treatment of malaria. The identity of the plant was confirmed by Mr. Patrick C. Mutiso of the Herbarium, Department of Biology, Faculty of Science and Technology, University of Nairobi, where a voucher specimen (DOO 2018/005) was deposited. The plant materials were air-dried under shade and ground to a powder using an electric grinder (Newtry 700g Electric Grinder Spice Mill 2400W, Guangdong, China).

### 4.2. Preparation of Test Extracts and Drugs

The roots, stems, and leaves of *S. longipedunculata* were extracted using methanol by percolation as earlier described [21]. Each of the plant extracts was dissolved in 99.5% dimethyl sulfoxide (DMSO) (Sigma-Aldrich, St. Louis, Germany) to make a concentration of 50,000 ng/mL then diluted in a complete Roswell Park Memorial Institute (RPMI 1640) (Gibco, New York, NY, USA) cell culture medium.

#### Reference *P. falciparum* Strains and Reference Antimalarial Drugs

The following cells were obtained through BEI Resources, NIAID, NIH: *P. falciparum*, Strain D6, MRA-285, Strain DD2, MRA-150, donated by David Walliker, and Strain W2, MRA-157 donated by Dennis E. Kyle.

The reference antimalarials drugs artemether, lumefantrine, piperaquine, dihydroartemisinin, chloroquine diphosphate, and Quinine were donated by the WorldWide Antimalarial Resistance Network (WWARN) External Quality Assurance Programme, Bangkok, Thailand [30].

The antimalarial drugs, obtained from WWARN, were dissolved in 99.5% DMSO (Sigma-Aldrich) and then diluted by complete Roswell Park Memorial Institute (RPMI 1640) (Invitrogen Inc.) cell culture medium to obtain starting concentrations of 200 ng/mL for artemether, lumefantrine, and dihydroartemisinin, piperaquine 500 ng/mL, chloroquine 2000 ng/mL, and quinine 4000 ng/mL as the starting concentration.

### 4.3. Drug Combinations

At least 10 mL of each of the plant extract at working concentrations was prepared in 15 mL centrifuge tubes and vortexed to homogeneity. Similarly, 10 mL of each of the reference drugs was prepared. Combining two entities for example plant roots extract and artemether proceeded with vortexing of the working concentration of the two entities then transferring volumes proportionate to fixed plant roots extract: artemether ratios of, 4:1, 3:1, 1:1, 1:2, 1:3, and 1:4, volume/volume into new pre-labelled test tubes as previously described by Ohrt and co-workers [24]. Then, 300 uL working concentration of the plant extract as well as artemisinin was transferred to well 1A of row 1 and well 1B of row 2 of a 96-well plate as separate drugs respectively. Further, 300 uL of the fixed plant roots extract: artemether ratios were vortexed to homogeneity and transferred to well 1C to well 1H of the same 96-well plate. With the first wells of all the rows filled with drugs, 150 uL of the diluent, the complete RPMI 1640 cell culture medium was added to all the remaining wells on the plate. A two-fold serial dilution was attained by gently mixing components of well 1A to 1H by aspirating and dispensing five times then transferring 150 uL to wells 2A to 2H using 8-way multichannel pipettes. The transferred drug was similarly mixed with the diluent in wells 2A to 2H using an 8-way multichannel pipette prior to again transferring 150 uL to wells 3A to 3H. This was repeated across the plate to wells 11A to 11H therefore, attaining a range of dilution of 97.7–50,000 ng/mL for extracts; 0.2 to 200 ng/mL for artemether or lumefantrine alongside proportionate lowering of concentrations for fixed-ratio combinations. Final DMSO concentration was ≤0.0875% [31]. The same procedure was repeated for every pair of combining partners. The diluted drugs were frozen at −65 to −80 degrees centigrade for not more than two weeks. Prior to use, a plate was thawed at room temperature and 25 uL coated to an assay plate in readiness for initiation of an assay.

### 4.4. Antiplasmodial Activity Assays

#### 4.4.1. In Vitro Antiplasmodial Activity

The non-radioactive Malaria SYBR Green I assay technique was used with modifications [29] to determine a concentration that inhibits 50% of *P. falciparum* parasites (IC_50_). This method depicts the replication of parasites with accuracy by the use of a non-radioactive fluorescent DNA dye (SYBR Green I) [32]. Three laboratory strains of *P. falciparum*, chloroquine-resistant, mefloquine-sensitive, artemisinin sensitive Indochina (W2), mefloquine-resistant, chloroquine-sensitive Sierra Leone I (D6), and mefloquine-resistant, chloroquine-resistant Indochina (DD2) were used. These strains of *P. falciparum* were maintained by standard culture method to adapt to in vitro culture and raise parasitemia [33,34]. Erythrocytes with 3–10% parasitemia were lowered to 1% at 2% hematocrit and added to drug plates pre-coated with samples (Figure S5). The plates were incubated for 72 h at 37 °C in a gas mixture of 5% CO_2_, 5% O_2_, and 90% N_2_. The assay was terminated by the addition of lysis buffer containing SYBR Green I dye (1 × final concentration) on each plate then incubated in the dark for 24 h at room temperature.

#### 4.4.2. Ex Vivo Antiplasmodial Activity

For ex vivo assays, clinical isolates of *P. falciparum* collected (Figure S4) from consented malaria individuals presenting at Kombewa Sub-County Hospital and Kisumu County Hospital with uncomplicated malaria as earlier described [35], were tested within 6 h of collection. Isolates with parasitemia exceeding 1% *P. falciparum* were adjusted to 1% parasitemia and 2% hematocrit, before adding onto pre-dosed 96-well plates. Equally, the ex vivo assay plates were incubated for 72 h at 37 °C in a gas mixture of 5% CO_2_, 5% O_2_, and 90% N_2_. The assay was terminated by the addition of lysis buffer containing SYBR Green I dye (1 × final concentration) on each plate then incubated in the dark for 24 h at room temperature.

### 4.5. Molecular Characterization of Field Isolates

#### 4.5.1. DNA (Deoxyribonucleic Acid) Extraction

DNA was extracted from a whole blood aliquot of each sample using the QIAamp^®^ DNA mini kit (QIAGEN Sciences, Inc., Düsseldorf, Germany) according to the manufacturer’s protocol.

#### 4.5.2. Species Determination

This analysis was done for malaria diagnosis using genus-specific qPCR (quantitative Polymerase Chain Reaction) assay. It was also done to determine *P. falciparum* and other *Plasmodium* species in the field isolates as previously described [36]. Reaction master mix was added in each well of the plate apart from negative template control (nuclease-free water) and positive control (*P. falciparum* DNA). All samples that were positive for malaria were further characterized for species composition as earlier described [37]. 

#### 4.5.3. Single Nucleotide Polymorphism (SNP) Analysis

The DNA (deoxyribonucleic acid) from all samples was genotyped for single nucleotide polymorphisms (SNPs). First, conventional PCR was used to amplify a fragment of *Pfmdr* 1, *Pfcrt*, *Pfdhfr*, *Pfdhps,* and *Pfmrp* 1 genes on an Applied Biosystems’ GenAmp PCR system 9700 (Foster City, CA, USA). The primary reaction was done as earlier described [38]. The SNPs were then determined by amplification of a fragment of the respective gene as earlier described [38] using real-time PCR machine Applied Biosystems’ prism 7500 Fast real-time PCR system (Foster City, CA, USA).

### 4.6. Data Analysis

Fluorescence units (RFUs) of SYBR Green I dye in each well in the plates were determined using Tecan Genios^®^ Plus (Tecan, OR, USA) set at emission and excitation wavelengths of 535 nm and 485 nm, respectively. The gain was set at 60, integration time at 40 µs, and the number of flashes at 10. The RFUs readings (Tables S1 and S2) were used to determine the concentration causing 50% inhibitory concentration (IC_50_s) for each sample using GraphPad Prism^®^ 8.0 windows software (Graphpad Software, San Diego, CA, USA). Only samples with appropriate dose response curves were included in calculation of the IC_50_s (Figures S1–S3). At least four separate replicate assays were done for each sample (quadruplicate) and presented as mean ± standard deviation (mean IC_50_ ± SD).

Parasite growth inhibition for combinations was calculated to give a 50% fractional inhibition concentration (FIC_50_) plot for each combination. The sum FIC_50_ for each sample was calculated by dividing the IC_50_ of the drug when used in combination by the IC_50_ of the drug when used alone and adding with the IC_50_ of the extract when used in combination by the IC_50_ of the extract when used alone as shown below. Sum FIC_50_s were used to generate scatter plots that explain the potency of a given combination by plotting at least all the quadruplicate assays on one plane which are grouped into synergism (FIC_50_ < 1), additivity (FIC_50_ = 1), and antagonism (FIC_50_ > 1) [23]. Sum FIC_50_ for each extract-drug combination ratio was determined using the following equation:

$$\mathrm{SUM}{\mathrm{FIC}}_{50}\;=\;\frac{{\mathrm{IC}}_{\mathrm{50}}\;\mathrm{of}\;A\;\mathrm{in}\;\mathrm{combination}}{{\mathrm{IC}}_{50}\;\mathrm{of}\;A\;\mathrm{alone}}+\frac{{\mathrm{IC}}_{50}\;\mathrm{of}\;B\;\mathrm{in}\;\mathrm{combination}}{{\mathrm{IC}}_{50}\;\mathrm{of}\;B\;\mathrm{alone}}$$

where A represents a partner drug and B represents the respective extract in the combination. 

One way analysis of variance (ANOVA) was done using GraphPad Prism^®^ 8.0 windows software (Graphpad Software, San Diego, CA, USA) to determine whether mean sum FIC_50s_ of one combination were different from other combinations. Turkey’s test was done for multiple comparisons of means that were found to be significantly different. A significance level of 0.05 (at *p* < 0.05) was used.

## 5. Conclusions

Most combinations of artemether and lumefantrine each combined with roots, stems, and leaves of *S. longipedunculata* extracts showed synergism in both in vitro and ex vivo antiplasmodial activities. The roots extract was the most potent and was enhanced when in combination with artemether. Roots extract combinations showed utmost synergistic responses which were observed in scatter plots of roots extract-drug combinations. In vivo studies of *S. longipedunculata* extracts and antimalarial drug combination is recommended to validate the potential use of plant extract-drug combination in the treatment of malaria.

## Figures and Tables

**Table 1:** Mean IC_50_s values (ng/mL) of drugs and *S. longipedunculata* methanol extracts.

Drugs and MeOH Extracts	Ex Vivo	W2	D6	DD2
Root extracts	9.8 ± 1.3	1.4 ± 0.07	2.9 ± 0.5	NT
Stem extracts	45.5 ± 6.2	NT	14.6 ± 2.6	16.7 ± 5.3
Leaf extracts	13.1 ± 1.8	20.4 ± 4.7	18.6 ± 4.8	NT
Artemether	0.8 ± 0.5	1.4 ± 0.1	0.8 ± 0.2	1.7 ± 0.3
Lumefantrine	61.1 ± 21.9	33.8 ± 7.6	40.1 ± 11.1	65.0 ± 21.2
Mefloquine	37.2 ± 10.6	0.8 ± 0.2	1.5 ± 0.4	1.8 ± 0.6
Chloroquine	120.1 ± 14.1	207.1 ± 77.8	7.4 ± 1.5	9.6 ± 1.3

Number of replicates: 4; NT: not tested; MeOH: methanol; values are 50% inhibition concentration are means ± standard deviation.

**Table 2:** Mean sum FIC_50_ values for artemether and lumefantrine combinations with *S. longipedunculata* against W2 and DD2 strains of *P. falciparum*.

Fixed Ratios Drug:Extract	W2 (*p* = 0.0007, R^2^ = 0.5648)				DD2 (*p* = 0.0002, R^2^ = 0.6165)			
	ART-Roots	LU-Roots	ART-Leaves	LU-Leaves	ART-Roots	LU-Roots	ART-Stems	LU-Stems
4:1	1.801 ± 1.899	0.691 ± 0.253	1.291 ± 0.0898	0.651 ± 0.405	0.875 ± 0.158	0.829 ± 0.292	0.371 ± 0.293	0.531 ± 0.574
3:1	0.546 ± 0.695	1.08 ± 0.847	1.277 ± 0.625	0.649 ± 0.481	1.078 ± 0.577	0.588 ± 0.334	0.219 ± 0.082	0.306 ± 0.198
1:1	0.705 ± 0.83	0.711 ± 0.41	1.444 ± 0.898	0.542 ± 0.252	0.713 ± 0.366	0.655 ± 0.477	0.775 ± 0.451	0.483 ± 0.476
1:2	0.759 ± 0.436	0.946 ± 0.648	2.046 ± 2.392	0.262 ± 0.122	0.802 ± 0.211	0.831 ± 0.504	0.424 ± 0.296	0.272 ± 0.23
1:3	0.428 ± 0.222	0.448 ± 0.354	2.351 ± 1.945	0.275 ± 0.319	0.796 ± 0.322	0.302 ± 0.147	0.524 ± 0.333	0.344 ± 0.157
1:4	0.897 ± 0.614	0.629 ± 0.488	3.978 ± 4.028	0.354 ± 0.119	1.11 ± 0.089	0.57 ± 0.343	0.596 ± 0.364	0.32 ± 0.309

Number of replicates: 4; ART: artemether; LU: lumefantrine; W2: chloroquine-resistant, mefloquine sensitive; DD2: chloroquine-resistant, mefloquine resistant, strains of *P. falciparum*; FIC_50_: synergism < 1, additivity 1, antagonism > 1; values are 50% inhibition concentration means ± standard deviation.

**Table 3:** Mean sum FIC_50_ values for artemether and lumefantrine combinations with *S. longipedunculata* extracts against D6 strain of *P. falciparum*.

Fixed Ratios Drug:Extract	ART-Roots	ART-Stems	ART-Leaves	LU-Roots	LU-Stems	LU-Leaves
4:1	0.425 ± 0.347	0.9 ± 0.255	0.65 ± 0.383	0.677 ± 0.251	1.435 ± 0.892	0.292 ± 0.133
3:1	0.468 ± 0.278	0.892 ± 0.446	0.664 ± 0.28	0.916 ± 0.427	0.956 ± 0.335	0.856 ± 0.307
1:1	0.43 ± 0.536	0.787 ± 0.434	0.763 ± 0.443	0.684 ± 0.173	2.27 ± 1.762	0.554 ± 0.161
1:2	0.342 ± 0.333	0.763 ± 0.238	1.147 ± 0.688	0.631 ± 0.213	0.613 ± 0.549	0.936 ± 0.523
1:3	0.471 ± 0.377	0.534 ± 0.251	0.959 ± 0.577	0.735 ± 0.306	1.924 ± 1.497	1.379 ± 0.641
1:4	0.284 ± 0.159	0.678 ± 0.145	1.132 ± 0.595	0.785 ± 0.427	1.351 ± 1.03	1.993 ± 1.094

Number of replicates: 4; ART: artemether; LU: lumefantrine; D6: mefloquine-resistant, chloroquine-sensitive strain of *P. falciparum*; FIC_50_: synergism < 1, additivity 1, antagonism > 1; values are 50% inhibition concentration means ± standard deviation.

**Table 4:** Mean sum FIC_50_ values for artemether and lumefantrine combinations with *S. longipedunculata* against field isolates ex vivo.

Fixed Ratios Drug:Extract	ART-Roots	LU-Roots	ART-Stems	LU-Stems	ART-Leaves	LU-Leaves
4:1	0.665 ± 0.294	0.548 ± 0.362	0.584 ± 0.336	0.765 ± 0.169	1.265 ± 0.32	0.791 ± 0.449
3:1	0.505 ± 0.426	3.179 ± 2.546	0.771 ± 0.514	0.483 ± 0.187	1.124 ± 0.313	0.365 ± 0.178
1:1	0.585 ± 0.39	0.86 ± 0.815	0.685 ± 0.066	1.168 ± 1.17	0.638 ± 0.276	0.72 ± 0.324
1:2	0.988 ± 0.464	0.916 ± 0.536	0.574 ± 0.149	0.985 ± 0.849	0.74 ± 0.343	1.149 ± 0.654
1:3	0.973 ± 0.536	0.352 ± 0.365	0.636 ± 0.093	0.752 ± 0.627	0.289 ± 0.155	0.88 ± 0.637
1:4	1.111 ± 0.664	0.526 ± 0.775	0.683 ± 0.425	0.6894 ± 0.28	0.174 ± 0.032	1.044 ± 0.526

Number of replicates: 6; ART: artemether; LU: lumefantrine; FIC_50_: synergism < 1, additivity 1, antagonism > 1; values are 50% inhibition concentration means ± standard deviation.

**Table 5:** Mean of mean sum FIC_50_s values across all fixed ratios for artemether and lumefantrine combined with *S. longipedunculata* extracts against strains of *P. falciparum*.

	ART-Roots	ART-Stems	ART-Leaves	LU-Roots	LU-Stems	LU-Leaves
D6	0.403 ± 0.068 ^a^	0.756 ± 0.126 ^b^	0.886 ± 0.206	0.738 ± 0.093 ^c^	1.425 ± 0.555 ^a,b,c^	1.001 ± 0.556
W2	0.856 ± 0.448 ^a^	NT	2.065 ± 0.944 ^a,b,c^	0.75 ± 0.207 ^b^	NT	0.456 ± 0.165 ^c^
DD2	0.896 ± 0.148 ^a,b^	0.485 ± 0.176 ^a^	NT	0.629 ± 0.18	0.376 ± 0.096 ^b^	NT
Field isolates *	0.805 ± 0.229	0.656 ± 0.067	0.705 ± 0.398	1.064 ± 0.996	0.807 ± 0.218	0.825 ± 0.252

NT: not tested; ART: artemether; LU: lumefantrine; D6: mefloquine-resistant, chloroquine-sensitive; W2: chloroquine-resistant, mefloquine sensitive; DD2: chloroquine-resistant, mefloquine-resistant strains of *P. falciparum*; FIC_50_: synergism < 1, additivity 1, antagonism > 1; * *p* value is not statistically significant; values are 50% inhibition concentration means ± standard deviation; ^a,b,c^: Combinations with same superscript letters are significantly similar.

## Data Availability

Data is contained within the article and supplementary materials.

## Supplementary Materials

The following are available online at https://www.mdpi.com/article/10.3390/plants11010047/s1. Figure S1; Antiplasmodial activity dose-response curves of artemether combined with roots extract of *S. longipedunculata* against DD2 *P. falciparum* strain. Figure S2; Antiplasmodial activity dose-response curves of mefloquine (MQ) against field isolates ex vivo collected from Kisumu County and Kombewa hospitals. Figure S3; Antiplasmodial activity dose-response curves of MQ and CQ against *Plasmodium falciparum* strains (D6, W2, and DD2). Figure S4; Blood samples received from nearby hospitals for ex vivo testings. Figure S5; Red blood cells, drug-extract combinations, *P. falciparum* strains, and test media in 96-well plate. Table S1; Relative fluorescence units and sum FIC_50_s for artemether combined with leaves extract against D6 *Plasmodium falciparum* strain. Table S2; Relative fluorescence units and sum FIC_50_s for artemether combined with leaves extract against field isolate ex vivo. Table S3 (excel); Molecular analysis data.

## References

[1] World Health Organization (WHO) (2020). Malaria Report.

[2] Zaw M.T., Emran N.A., Lin Z. (2018). Updates on k13 mutant alleles for artemisinin resistance in Plasmodium falciparum. J. Microbiol. Immunol. Infect..

[3] Menard D., Dondorp A. (2017). Antimalarial drug resistance: A threat to malaria elimination. Cold Spring Harb. Perspect. Med..

[4] Saifi M.A., Beg T., Harrath A.H., Altayalan F.S.H., Qurais S.A. (2013). Antimalarial drugs: Mode of action and status of resistance. Afr. J. Pharm. Pharmacol..

[5] Tse E.G., Korsik M., Todd M.H. (2019). The past, present and future of anti-malarial medicines. Malar. J..

[6] Lu F., He X.L., Richard C., Cao J. (2019). A brief history of artemisinin: Modes of action and mechanisms of resistance. Chin. J. Nat. Med..

[7] Smith C.S., Aerts A., Saunderson P., Kawuma J., Kita E., Virmond M. (2017). Review Multidrug therapy for leprosy: A game changer on the path to elimination. Lancet Infect. Dis..

[8] Kerantzas C.A., William R.J. (2017). Origins of Combination Therapy for Tuberculosis: Lessons for Future Antimicrobial Development and Application. Am. Soc. Microbiol..

[9] Chesney M.A., Morin M., Sherr L. (2000). Adherence to HIV Combination Therapy. Soc. Sci. Med..

[10] World Health Organization (WHO) (2021). Guidelines for Malaria.

[11] Bhatt S., Weiss D.J., Cameron E., Bisanzio D., Mappin B., Dalrymple U., Battle K.E., Moyes C.L., Henry A., Eckhoff P.A. (2015). The effect of malaria control on Plasmodium falciparum in Africa between 2000 and 2015. Nature.

[12] Banda C.G., Chaponda M., Mukaka M., Mulenga M., Hachizovu S., Kabuya J.B., Mulenga J., Sikalima J., Phiri L.K., Terlouw D.J. (2019). Efficacy and safety of artemether–lumefantrine as treatment for Plasmodium falciparum uncomplicated malaria in adult patients on efavirenz-based antiretroviral therapy in Zambia: An open label non-randomized interventional trial. Malar. J..

[13] Kaur R., Kaur H. (2017). Plant Derived Antimalarial Agents. J. Med. Plants Stud..

[14] Mongalo N.I., McGaw L.J., Finnie J.F., Staden J.V. (2015). Securidaca longipedunculata Fresen (Polygalaceae): A review of its ethnomedicinal uses, phytochemistry, pharmacological properties and toxicology. J. Ethnopharmacol..

[15] Ochora D.O., Saifudin F.D., Nguta J.M., Akunda E.M. (2014). Antimalarial activity and acute toxicity of four plants traditionally used in treatment of malaria in Msambweni District of Kenya. Eur. Int. J. Sci. Technol..

[16] Hamill F.A., Apio S., Mubiru N.K., Bukenya-Ziraba R., Mosango M., Maganyi O.W., Soejarto D.D. (2003). Traditional herbal drugs of Southern Uganda, II: Literature analysis and antimicrobial assays. J. Ethnopharmacol..

[17] Opio D., Andama E., Kureh G. (2018). Ethnobotanical Survey of Antimalarial Plants in Areas of: Abukamola, Angeta, Oculokori and Omarari of Alebtong District in Northern Uganda. Eur. J. Med. Plants.

[18] Joseph C.C., Moshi M.J., Sempombe J., Nkunya M.H.H. (2006). (4-methoxy-benzo[1,3]dioxol-5-yl)-phenylmethanone: An antibacterial benzophenone from Securidaca longepedunculata. Afr. J. Tradit. Complement. Altern. Med..

[19] Nadembega P., Boussim J.I., Nikiema J.B., Poli F., Antognoni F. (2011). Medicinal plants in Baskoure, Kourittenga Province, Burkina Faso: An ethnobotanical study. J. Ethnopharmacol..

[20] Mustapha A.A. (2013). Ethno-medico-botanical uses of Securidaca longepedunculata Fresen (family-polygalaceae) from Keffi local government, Nasarawa state, Nigeria. J. Nat. Remedies.

[21] Ochora D.O., Kakudidi E., Namukobe J., Heydenreich M., Coghi P., Yang L.J., Mwakio E.W., Andagalu B., Roth A., Akala H.M. (2021). A new benzophenone, and the antiplasmodial activities of the constituents of Securidaca longipedunculata fresen (Polygalaceae). Nat. Prod. Res..

[22] Ocloo A., Okpattah W.E., Quasie W.O., Sakyiamah M.M., Okine K.N. (2014). Concurrent administration of aqueous extract of Cryptolepis sanguinolenta reduces the effectiveness of ART against Plasmodium berghei in rats. J. Appl. Pharm. Sci..

[23] Akala H.M., Waters C.N., Yenesew A., Wanjala C., Akenga T.A. (2010). In vitro antiplasmodial and cyclin-dependent protein kinase (pfmrk) inhibitory activities of selected flavonoids in combination with chloroquine (CQ) and artemisinin. Res. Pharm. Biotechnol..

[24] Ohrt C., Willingmyre G.D., Lee P., Knirsch C., Milhous W. (2002). Assessment of Azithromycin in Combination with Other Antimalarial Drugs against Plasmodium falciparum In Vitro. Antimicrob. Agents Chemother..

[25] Gathirwa J.W., Rukunga G.M., Njagi E.N.M., Omar S.A., Mwitari P.G., Guantai A.N., Tolo F.M., Kimani C.W., Muthaura C.N., Kirira P.G. (2008). The in vitro anti-plasmodial and in vivo anti-malarial efficacy of combinations of some medicinal plants used traditionally for treatment of malaria by the Meru community in Kenya. J. Ethnopharmacol..

[26] Adegbolagun O.M., Emikpe B.O., Woranola I.O., Ogunremi Y. (2013). Synergistic effect of aqueous extract of Telfaria occidentalis on the biological activities of artesunate in Plasmodium berghei infected mice. Afr. Health Sci..

[27] Ukwe C.V., Ekwunife O.I., Epueke E.A., Ubaka C.M. (2010). Antimalarial activity of Ageratum conyzoides in combination with chloroquine and artesunate. Asian Pac. J. Trop. Med..

[28] Mukungu N., Abuga K., Okalebo F., Ingwela R., Mwangi J. (2016). Medicinal plants used for management of malaria among the Luhya community of Kakamega East sub-County, Kenya. J. Ethnopharmacol..

[29] Erhirhie E.O., Ikegbune C., Okeke A.I., Onwuzuligbo C.C., Madubuogwu N.U., Chukwudulue U.M., Okonkwo O.B. (2021). Antimalarial herbal drugs: A review of their interactions with conventional antimalarial drugs. Clin. Phytosci..

[30] Lourens C., Watkins W.M., Barnes K.I., Sibley C.H., Guerin P.J., White N.J., Lindegardh N. (2010). Implementation of a reference standard and proficiency testing programme by the World Wide Antimalarial Resistance Network (WWARN). Malar. J..

[31] Yenesew A., Akala H.M., Twinomuhwezi H., Chepkirui C., Irungu B.N., Eyase F.L., Kamatenesi-Mugisha M., Kiremire B.T., Johnson J.D., Waters N.C. (2012). The antiplasmodial and radical scavenging activities of flavonoids of Erythrina burttii. Acta Trop..

[32] Smilkstein M., Sriwilaijaroen N., Kelly J.X., Wilairat P., Riscoe M. (2004). Simple and Inexpensive Fluorescence-Based Technique for High-Throughput Antimalarial Drug Screening. Antimicrob. Agents Chemother..

[33] Johnson J.D., Dennull R.A., Gerena L., Lopez-Sanchez M., Roncal N.E., Waters N.C. (2007). Assessment and continued validation of the malaria SYBR Green I-based fluorescence assay for use in malaria drug screening. Antimicrob. Agents Chemother..

[34] Chulay J.D., Haynes J.D., Diggs C.L. (1983). Plasmodium falciparum: Assessment of in Vitro Growth by [3H] Hypoxanthine Incorporation. Exp. Para..

[35] Akala H.M., Eyase F.L., Cheruiyot A.C., Omondi A.A., Ogutu B.R., Waters N.C., Johnson J.D., Polhemus M.E., Schnabel D.C., Walsh D.S. (2011). Antimalarial drug sensitivity profile of western Kenya Plasmodium falciparum field isolates determined by a SYBR green I in vitro assay and molecular analysis. Am. J. Trop. Med. Hyg..

[36] Kamau E., Tolbert L.S., Kortepeter L., Pratt M., Nyakoe N., Muringo L., Ogutu B., Waitumbi J.N., Ockenhouse C.F. (2011). Development of a highly sensitive genus-specific quantitative reverse transcriptase real-time PCR assay for detection and quantitation of plasmodium by amplifying RNA and DNA of the 18S rRNA genes. J. Clin. Microbiol..

[37] Akala H.M., Watson O.J., Mitei K.K., Juma D.W., Verity R., Opot B.H., Okoth R.O., Chemwor G.C., Juma J.A., Mwakio E.W. (2021). Plasmodium interspecies interactions during a period of increasing prevalence of Plasmodium ovale in symptomatic individuals seeking treatment: An observational study. Lancet Microbe.

[38] Maraka M., Akala H.M., Amolo A.S., Juma D., Omariba D., Cheruiyot A., Opot B., Okudo C.O., Mwakio E., Chemwor G. (2020). A seven-year surveillance of epidemiology of malaria reveals travel and gender are the key drivers of dispersion of drug resistant genotypes in Kenya. Peer J..

