# Supplementary material for: Synergism in Antiplasmodial Activities of Artemether and Lumefantrine in Combination with Securidaca longipedunculata Fresen (Polygalaceae)
Source: Plants (Basel). 2021 Dec 24;11(1):47. doi: 10.3390/plants11010047 (PMC8747340; doi:10.3390/plants11010047)
Supplement: Supplementary file 1 [file plants-11-00047-s001.zip › plants-1414099-supplementary-tables and figures.pdf]

SELECTED ANTIPLASMODIAL ACTIVITY DOSE RESPONSE CURVES

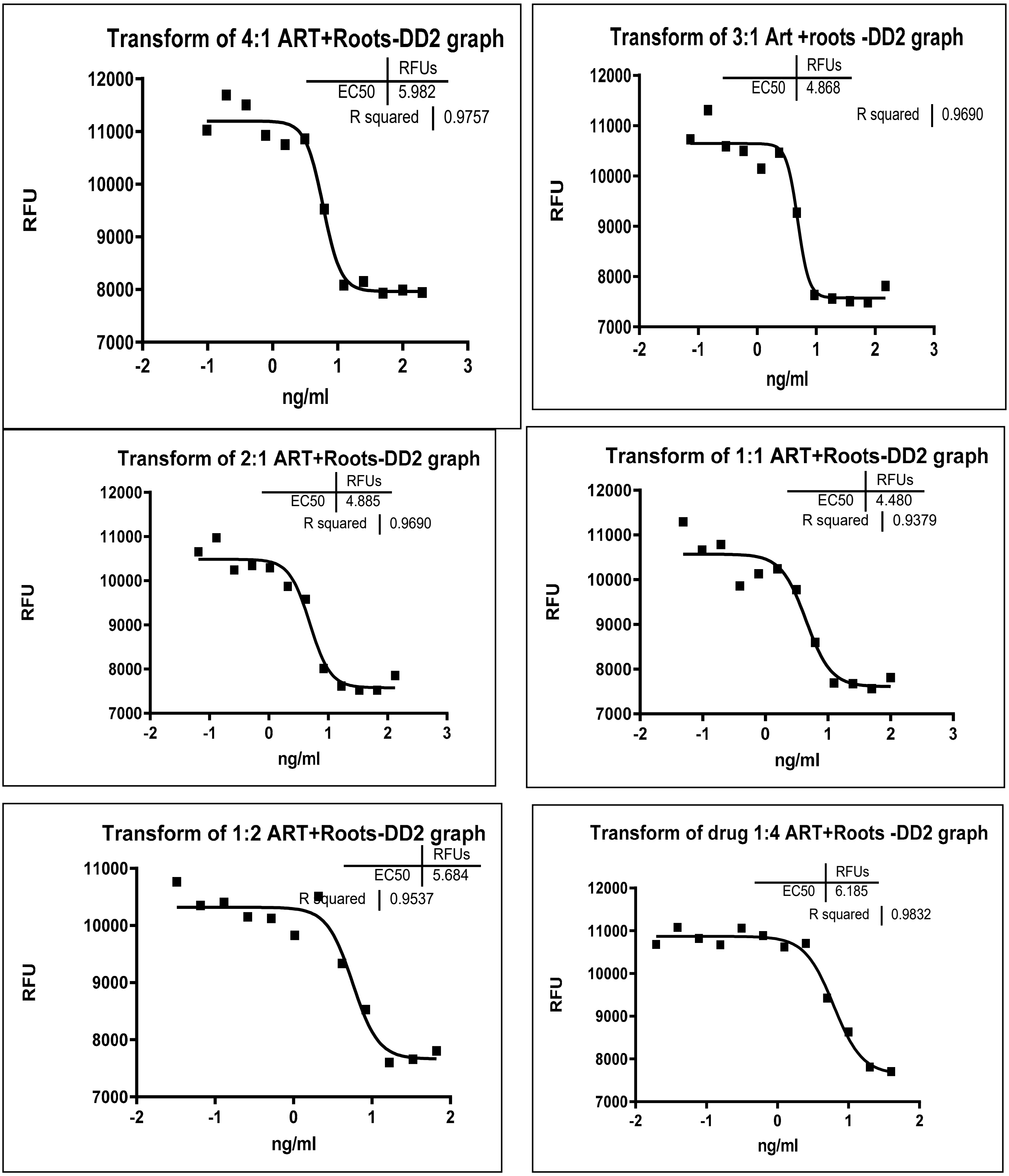

Figure S1: Antiplasmodial activity dose response curves of Artemether combined with roots extract of *S. longipedunculata* against DD2 *P. falciparum* strain.

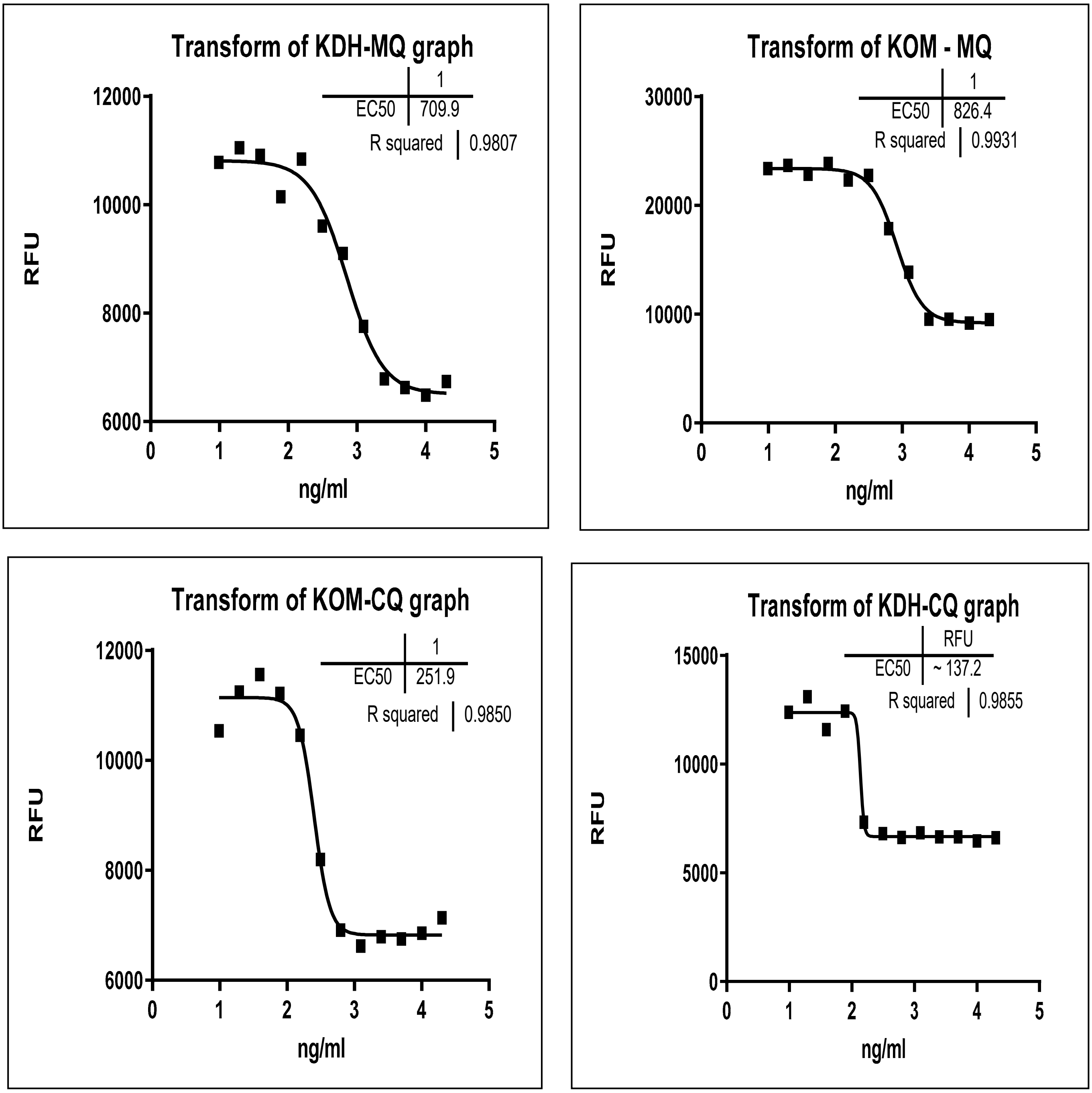

Figure S2: Antiplasmodial activity dose response curves of Mefloquine (MQ) against field isolates *ex vivo* collected from Kisumu County and Kombewa hospitals.

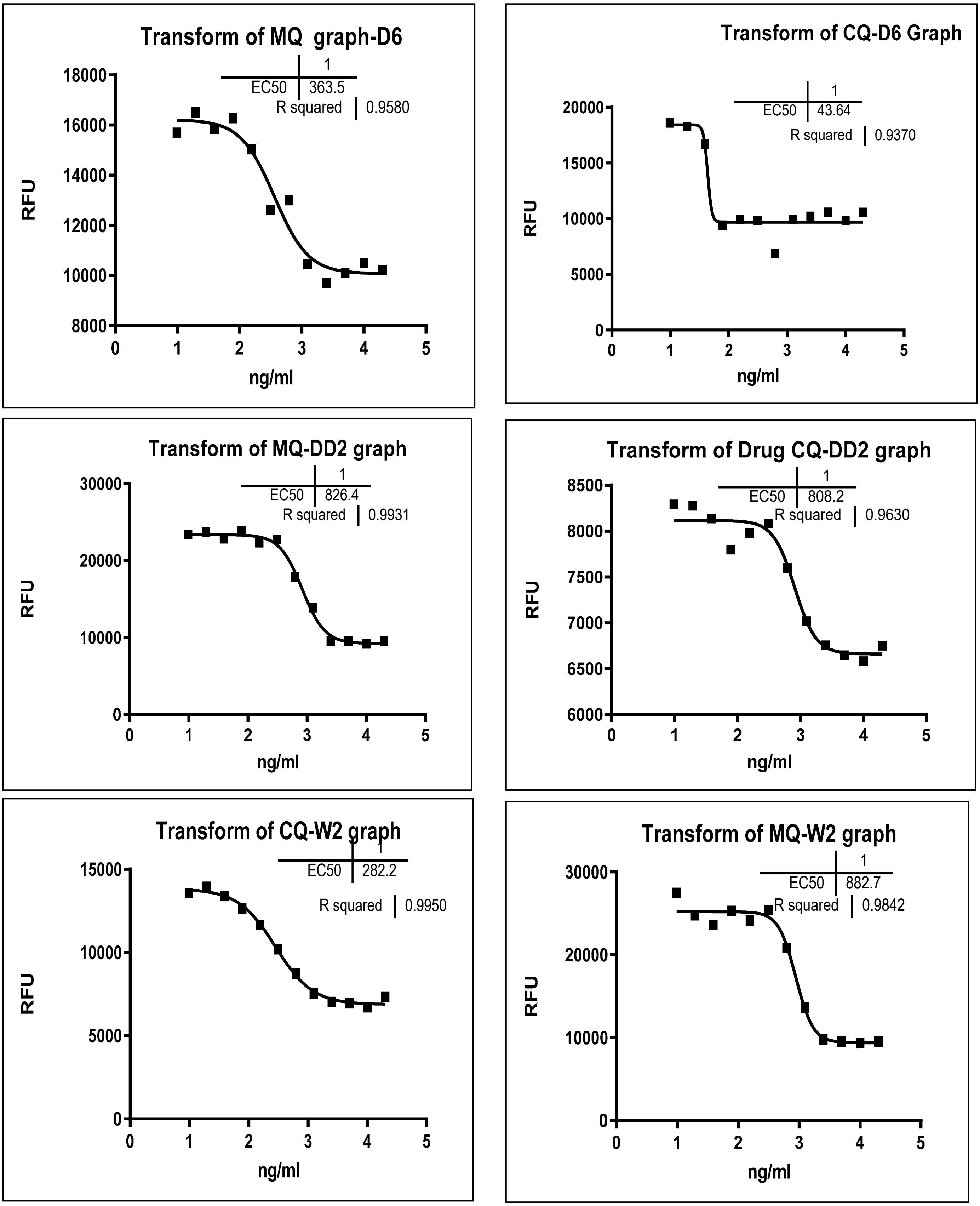

Figure S3: Antiplasmodial activity dose response curves of MQ and CQ against *Plasmodium falciparum* strains (D6, W2 and DD2).

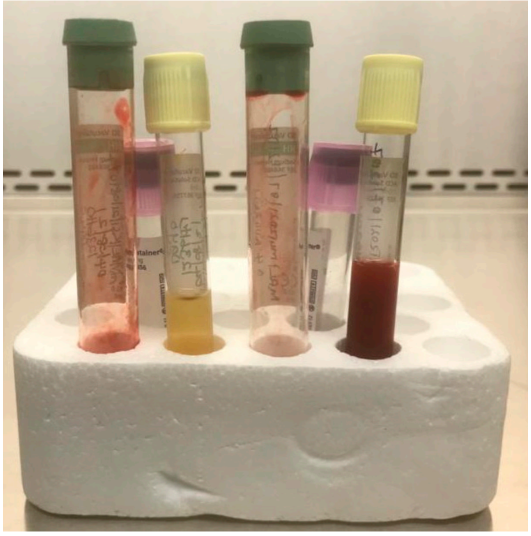

Figure S4: Blood samples receieved from nearby hospitals for immediate *ex vivo* testings.

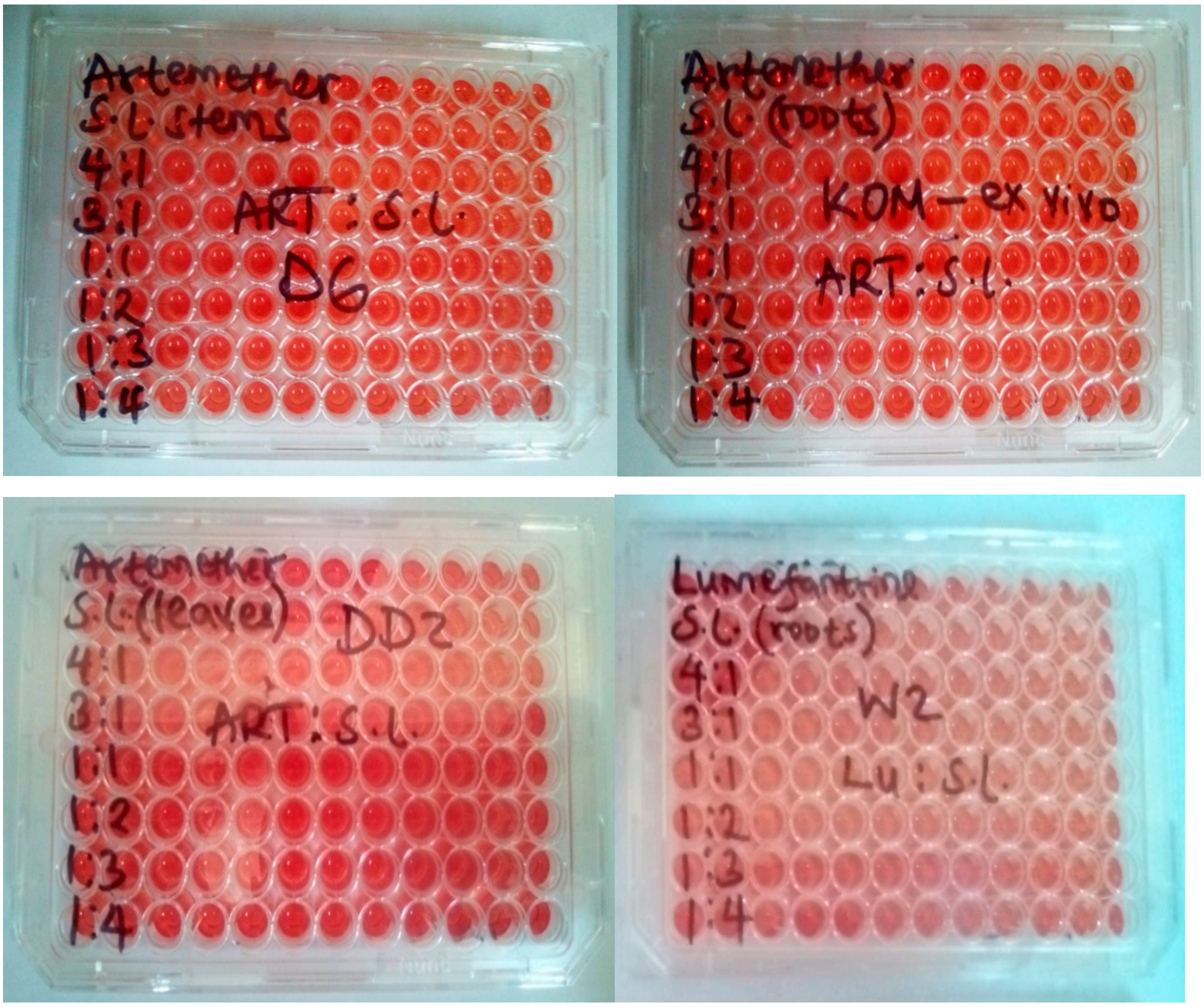

Figure S5: Red blood cells, drug-extract combinations, *P. faciparum* strains and test media in 96-well plates.

SELECTED RELATIVE FREQUENCY UNITS (RFUs) AND SUM 50% FRACTIONAL INHIBITION CONCENTRATIONS (FIC_{50}s)

Table S1: Relative fluorescence units and sum FIC_{50}s for artemether combined with leaves extract against D6 *Plasmodium falciparum* strain.

GENios Plus; Serial number: 610000002; Firmware: V 6.03 13_07_2005 Genios; XFLUOR4 Version: V 4.51												**Artemether + leaves extracts against D6**			
Date:					27-12-20										
Time:					09:59			Fixed ratic	FIC Art	FIC MeOH leaves			**FIC50 A**	**FIC50 B**	**SUM FIC50**
								A	42.98	0			1	0	
Measurement mode:					Fluorescence Bottom			4:01	25.15	2106			0.58516	0.04768	0.63284
Excitation wavelength:					485	nm		3:01	24.15	3014			0.56189	0.06824	0.63013
Emission wavelength:					535	nm		1:01	24.58	6144			0.57189	0.13911	0.71101
Gain (Manual):					60			1:02	31.74	15870			0.73848	0.35933	1.09781
Number of flashes:					10			1:03	19.92	14941			0.46347	0.33829	0.80176
Lag time:					0	µs		1:04	21.68	21683			0.50442	0.49094	0.99536
Integration time:					40	µs		B	0	44166			0	1	
Plate definition file:					NUN96ft.pdf							SUMFIC5(	3.42531	1.4436	**4.86891**
Rawdata					Temperature: 24.7 °C										
<>	1	2	3	4	5	6	7	8	9	10	11	12			
A	800	819	951	1383	1312	1358	1305	1457	1429	1428	1265	1573			
B	1079	1187	1274	1335	1299	1338	1313	1326	1346	1328	1266	1463			
C	805	818	966	1150	1309	1337	1312	1364	1316	1341	1301	1500			
D	806	823	992	1148	1284	1330	1297	1364	1356	1380	1272	1445			
E	859	910	1069	1219	1251	1308	1330	1375	1206	1314	1266	1432			
F	891	1054	1211	1305	1323	1315	1275	1326	1268	1324	1254	1451			
G	866	1000	1223	1306	1308	1263	1381	1348	1299	1351	1290	1386			
H	885	1104	1275	1358	1341	1352	1327	1280	1283	1275	1350	1509			

Table S2: Relative fluorescence units and sum FIC_{50}s for artemether combined with leaves extract against field isolate *ex vivo*.

GENios Plus; Serial number: 610000002; Firmware: V 6.03 13_07_2005 Genios; XFLUOR4 Version: V 4.51															
Date:					31-1-21						Artemether +leaves extracts against KDH-01/195				
Time:					20:25										
								Fixed Ra	FIC50 AR	FIC50 Leaves			FIC50 A	FIC50 B	SUM FIC50
Measurement mode:					Fluorescence Bottom			A	49.36	0			1	0	
Excitation wavelength:					485	nm		4:01	37.51	3109			0.759927	0.151799	0.911726
Emission wavelength:					535	nm		3:01	33.76	4188			0.683955	0.204482	0.888437
Gain (Manual):					60			1:01	13.12	3279			0.265802	0.1601	0.425902
Number of flashes:					10			1:02	10.34	5173			0.209481	0.252576	0.462057
Lag time:					0	µs		1:03	3.543	2657			0.071779	0.12973	0.201509
Integration time:					40	µs		1:04	3.127	3127			0.063351	0.152678	0.216029
Plate definition file:					NUN96ft.pdf			B	0	20481			0	1	
												SUMFIC5	2.054295	1.051365	**3.10566**
Rawdata					Temperature: 26.8 °C										
<>	1	2	3	4	5	6	7	8	9	10	11	12			
A	5766	2416	3162	3800	3586	4477	5850	5507	5418	4877	6828	4902			
B	3792	4161	5418	5610	5523	6073	7349	5995	6615	5018	6549	4807			
C	2166	2412	3914	5762	5330	5985	4954	5909	6777	5348	5148	4896			
D	2143	2153	4181	6013	5757	6348	5132	5903	6408	5076	7087	4990			
E	2082	2131	3313	3882	5012	6418	5990	6103	6665	5973	5742	5162			
F	2089	2183	3054	4269	5815	5959	5558	5835	6957	5279	5497	5278			
G	2143	2199	3030	2919	4474	6280	5218	5900	7858	5590	6193	5225			
H	2168	2244	3459	2975	4525	5858	5268	5981	6876	5647	5422	4787			
